# Schinzel-Giedion syndrome: a case with sacrococcygeal teratoma and cor-triatriatum dexter

**DOI:** 10.11604/pamj.2017.26.30.11525

**Published:** 2017-01-23

**Authors:** Lofty-John Anyanwu, Aminu Mohammad, Habeeb Muhammad, Ibrahim Aliyu, Lawal Abdullahi, Aliyu Farinyaro, Abdulkarim Iya

**Affiliations:** 1 Paediatric Surgery Unit, Department of Surgery, Aminu Kano Teaching Hospital and Bayero University Kano, Nigeria; 2 Department of Anaesthesiology and Intensive Care, Aminu Kano Teaching Hospital Kano, Nigeria; 3 Paediatric Cardiology Unit, Department of Paediatrics Aminu Kano Teaching Hospital and Bayero University Kano, Nigeria

**Keywords:** Schinzel-Giedion syndrome, sacrococcygeal teratoma, cor-triatriatum dexter, midfacial hypoplasia, embryonic tumors

## Abstract

Schinzel-Giedion syndrome which is associated with midfacial hypoplasia and coarse dysmorphic features is a multiple congenital malformation syndrome. It is believed that risk of malignancy may be a component of the anomaly. We report herein a case of a 16 months old boy with SGS associated with sacrococcygeal teratoma and cor-triatriatum dexter. Histopathology report of the excised sacrococcygeal mass showed no malignant foci. He was however lost to follow up in the second week post-operation.

## Introduction

Schinzel and Giedion in 1978 described a new syndrome in two siblings with severe midface retraction, multiple skull and skeletal anomalies, and cardiac and renal malformations [1]. The midface retraction which is pathognomonic consists of shallow orbits and midface hypoplasia with resulting prominence of the forehead, large fontanelles and widely patent cranial sutures, particularly the metopic suture [2]. Although recessive inheritance is likely in Schinzel-Giedion syndrome (SGS), the exact mode of inheritance remains unclear [3]. Alternative hypotheses such as an autosomal dominant mutation, microdeletion, or microduplication with the few recurrences being explained by parental gonadal mosaicism, have also been considered [2]. Recent evidence suggests that de novo mutations of SETBP1 cause SGS [4, 5]. Since a high frequency (15%) of embryonic tumors has been noted in the reported cases of SGS, it is thus being considered that a risk of malignancy could be a component of the syndrome [2, 3, 6]. Details of a new case of SGS with sacrococcygeal teratoma and cor-triatriatum dexter are provided in this report, as well as the challenges faced in his management.

## Patient and observation

**Figure 1:**
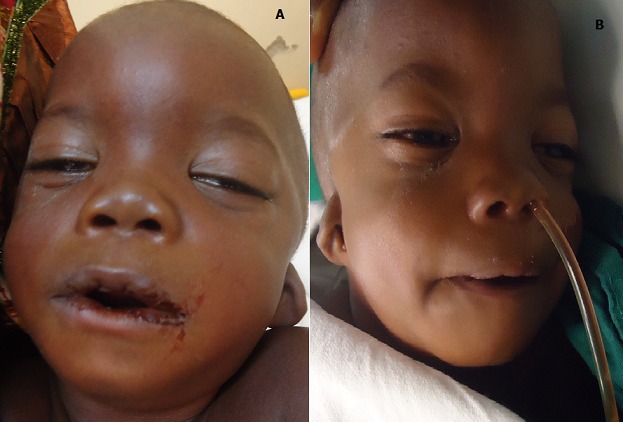
Frontal view (A) and right lateral view (B) of a child with Schinzel-Giedion syndrome. Note the tall fore head, hypertelorism and microtic protruded ear lobes

**Figure 2:**
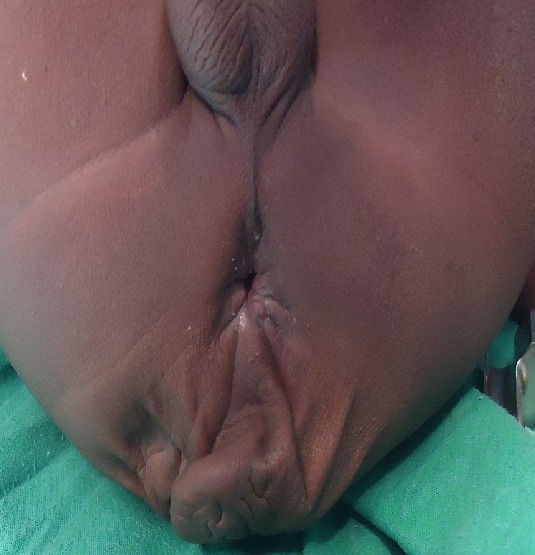
Sacrococcygeal mass in the same patient

**Figure 3:**
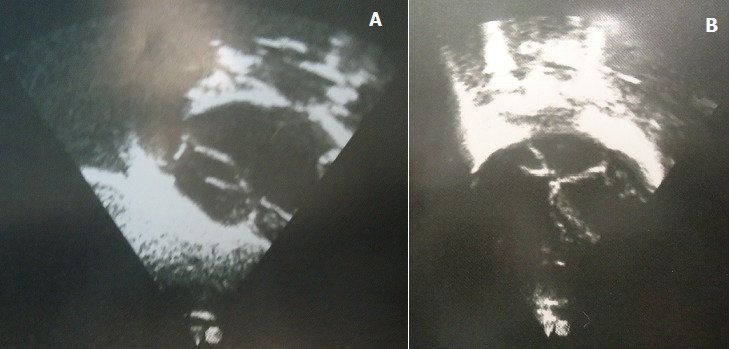
(A) subcostal view and (B) apical four chamber view of transthoracic echocardiography of the patient, showing a thick band of echogenic tissue dividing the right atrium into 2 unequal halves

The patient who was a 16 month old boy was seen in our out-patient clinic on account of multiple congenital anomalies which were noticed at birth. His mother also complained of his inability to open his mouth wide when crying or feeding. His parents were not blood related. He was the fifth child of his mother, and there was no family history of the birth of a child with a congenital anomaly. His pregnancy birth and neonatal history were uneventful. On examination, he was irritable, afebrile, anicteric, acyanosed, normotonic and not in any obvious respiratory distress. His pulse rate was 118 per minute, which was regular and of normal volume. Heart sounds 1 and 2 were heard on auscultation, with no murmurs. He had clear lung fields with good air entry bilaterally. There were no significant findings on abdominal examination, and he had normal external male genitalia. A facial examination showed he had a coarse facial appearance with a flat nasal bridge, hypertelorism, low set microtic ears with protruding ear lobules and a short upturned nose (Figure 1). He had an inter-incisal distance of about 13mm at maximal mouth opening. He had a back swelling measuring about 15cm by 25cm (Figure 2) in the lower sacral region, which was sessile, not tender and had no differential warmth. It had an irregular surface, it was soft and had none distinct edges. The swelling had no underlying bony defect, and no pelvic extension was noted on digital rectal examination. His limbs were normal and his serum alpha-fetoprotein level was 31mcg/L (normal 0-5mcg/L). A two dimensional transthoracic echocardiography was done (Figure 3), which showed situs solitus of the atria and viscera, with atrioventricular and ventriculoarterial concordance. The mitral, tricuspid, pulmonary and aortic valves were normal, and chamber walls showed good contractility. There was no atrial septal defect (ASD), patent ductus arteriosus (PDA) nor ventricular septal defect (VSD) seen. A thick band of echogenic tissue separating the right atrium into 2 unequal halves with a wide eccentric aperture communicating both chambers was seen. Abdomino-pelvic ultrsonography showed normal right and left kidneys.

Based on his clinical features and investigation results, a diagnosis of Schinzel-Giedion syndrome with sacrococcygeal teratoma (SCT) and an asymptomatic cor-triatriatum dexter was made. Genetic analysis was however not done in this patient, as we currently do not have facilities for such a test in our hospital. Due to the documented risk of malignancy in embryonic tumors in SGS [2, 3, 6], a decision for an early excision of the sacrococcygeal mass was made. At induction of anaesthesia, difficulties were encountered at attempts to pass an endotracheal tube due to the inability to open the patient’s mouth wide enough for the placement of a laryngoscope. Bilateral congenital temporo-mandibular joint (TMJ) ankylosis was diagnosed, and attempts at endotracheal intubation were abandoned. Since we did not have facilities for fibre optic laryngeal intubation, a laryngeal mask airway (LMA) was inserted and naso-gastric intubation for gastric decompression done. The procedure of SCT excision was performed with the patient in the left lateral position; instead of the conventional prone jack-knife position. His post-operative recovery was uneventful and he was discharged to the outpatient clinic for follow up on the tenth day post operation. The histopathology report of the excised tumour showed a solid mass lined externally by unremarkable epidermis. The parenchyma demonstrated smooth and skeletal muscles, adipose tissues, cartilage and glandular tissue with no malignant foci. During his first follow up visit by the third week post operation, he was referred to the Maxillofacial surgery unit on account of his TMJ ankylosis. He was however subsequently lost to follow up.

## Discussion

Schinzel-Giedion syndrome (SGS) is a rare multiple malformation syndrome with a high prevalence of tumors [4]. The commonly reported features of the syndrome include severe midface retraction, multiple skull anomalies, congenital heart defects, hydronephrosis, clubfeet and hypertrichosis [2, 3 ]. The facial appearance is often described as being coarse, with hypertelorism, flat nasal bridge, anteverted nares and low set ears with protruding lobules [2]. The presence of these features in our patient formed the bases for the diagnosis. Gain of function/dominant negative mutations in SETBP1, a gene encoding the oncogene-binding protein, SET-binding protein 1, has been reported to be the causative genetic defect in SGS [7]. Although little is known about the function of SETBP1, it might be associated with bone development and specific childhood tumors [4]. SETBP1 overexpression is believed to promote leukemogenesis by enhancing full length SET protein and then impairing the phosphatise activity of the tumor suppressor PP2A through the formation of a SETBP1-SET-PP2A complex [5]. In a review of 46 reported cases of SGS by McPherson, 7(15%) had childhood tumors including 3 sacrococcygeal teratomas, 2 primitive neurectodermal tumors arising in the sacral area, 1 hepatoblastoma, and 1 Wilm’s tumor arising in a multicystic dysplastic kidney [2 ]. Our patient had a sacrococcygeal teratoma. The most important systemic involvements other than the skeletal system are cardiac defects, renal defects in the form of hydronephrosis, with central nervous (CNS) malformations being reported occasionally, although no generally accepted diagnostic guidelines exist [2, 8]. Lehman et al., proposed that a clinical diagnosis may be made in this syndrome by identifying the facial phenotype, including prominent forehead, midface retraction, and short, upturned nose, plus one of either of the two other major distinguishing features: typical skeletal abnormalities or hydronephrosis [9]. Our patient had the characteristic facial phenotype and a skeletal deformity i.e. congenital ankylosis of both temporomandibular joints, with an inter-incisal distance of about 13mm at maximal mouth opening. Abdomino-pelvic ultrasonography in our patient revealed normal kidneys. Intravenous urography was however not done for the patient. Alavi et al., had earlier reported similar findings in their patient [8]. The natural history of this condition is severe growth retardation and profound mental deficiencies, with seizures of varying types, often including infantile spasms being reported in most patients [2, 8]. Our patient was apparently normal on CNS evaluation, however no neuro-imaging studies were done on him. Since he was lost to follow up, his disease progression could not be characterised. About one out of three of patients with SGS have been documented to have congenital cardiac defects [2]. Two-dimensional transthoracic echocardiography (Figure 3) in our patient showed he had cor-triatriatum dexter. He was however asymptomatic on cardiovascular evaluation. Cor-triatriatum dexter is an extremely rare congenital heart defect which represents approximately 0.1% of all congenital heart anomalies [10, 11]. In this anomaly, the right atrium is divided into two by a septum, resulting in three atrial chambers. Cor-triatriatum dexter is believed to result from lack of normal regression of the embryonic right valve of the sinus venosus [10]. Mild septation of the right atrium is often asymptomatic and is mostly documented as an incidental finding during surgery to correct other cardiac abnormalities or during echocardiography, although more severe septation can cause right sided heart failure and elevated central venous pressure due to obstruction of the tricuspid valve, the right ventricular outflow tract, or the inferior vena cava [11]. The clinical features in SGS seem to overlap with those seen in children with peroxisomal disorders [7]. In the differential diagnosis of SGS, the coarse facial features seen in the children resemble that seen in children with metabolic diseases such as mucopolysaccharidosis, cretinism, and gangliosidosis [8]. There are many who believe that a metabolic abnormality is the most likely cause of SGS [4]. We were not able to do appropriate biochemical tests to exclude metabolic disorders in this patient. Treatment of patients with this condition is mainly symptomatic and directed at complications [2]. Regular follow up in these patients is thus mandatory.

## Conclusion

Although Schinzel- Giedion syndrome is a multiple congenital anomaly disorder, the risk of malignancy in this condition is high and potentially life threatening. We recommend a prompt excision and histopathological characterization of tumors in these patients as this could be life saving. It is important to remember that this syndrome could be a differential diagnosis for a child with an embryonic tumour and coarse dysmorphic features.

## References

[1] Schinzel A, Giedion A (1978). A syndrome of severe midface retraction, multiple skull anomalies, club feet, and cardiac and renal malformations in sibs. Am J Med Genet..

[2] McPherson E (2006). Schinzel-Giedion midface retraction syndrome. Atlas Genet Cytogenet Oncol Haematol..

[3] Sandri A, Manazza AD, Bertin D, Silengo M, Basso ME, Forni M, Madon E (2003). Schinzel-Giedion syndrome with sacrococcygeal teratoma. J Pediatr Hematol Oncol..

[4] Park KH, Hwang SH, Byun SY (2013). A case of Schinzel- Giedion syndrome. Neonatal Med..

[5] Cristobal I, Garcia-Orti L, Odero MD (2013). SETBP1 (SET binding protein 1). Atlas Genet Cytogenet Oncol Haematol..

[6] Matsumoto F, Tohda A, Shimada K, Okamoto N (2005). Malignant retroperitoneal tumor arising in a multicystic dysplastic kidney of a girl with Schinzel-Giedion syndrome. Int J Urol..

[7] Lestner JM, Chong WK, Offiiah A, Kefas J, Vandersteen AM (2012). Unusual neuroradiological features in Schinzel-Giedion syndrome: A novel case. Clin Dysmorphol..

[8] Alavi S, Kher A, Bharucha BA (1994). Schinzel-Giedion syndrome. Indian Pediatr..

[9] Lehman AM, McFadden D, Pugash D, Sangha K, Gibson WT, Patel MS (2008). Schinzel-Giedion syndrome: Report of splenopancreatic fusion and proposed diagnostic criteria. Am J Med Genet A..

[10] Alboliras ET, Edwards WD, Driscoll DJ, Seward JB (1987). Cor triatriatum dexter: Two- dimensional echocardiographic diagnosis. J Am Coll Cardiol..

[11] Meher BK, Pradeep S, Das L, Tripathy P (2013). Cor-triatriatum dexter with pulmonary hypertension in a neonate. Open Journal of Pediatrics..

